# ER-mitochondria contact sites; a multifaceted factory for Ca^2+^ signaling and lipid transport 

**DOI:** 10.3389/fcell.2022.988014

**Published:** 2022-08-16

**Authors:** Maria Livia Sassano, Blanca Felipe-Abrio, Patrizia Agostinis

**Affiliations:** ^1^ Cell Death Research and Therapy Group, Department of Cellular and Molecular Medicine, Leuven, Belgium; ^2^ VIB Center for Cancer Biology, Leuven, Belgium

**Keywords:** ER-mitochondria contact sites, lipids, lipid transfer protein, Ca^2+^ signaling, cancer

## Abstract

Membrane contact sites (MCS) between organelles of eukaryotic cells provide structural integrity and promote organelle homeostasis by facilitating intracellular signaling, exchange of ions, metabolites and lipids and membrane dynamics. Cataloguing MCS revolutionized our understanding of the structural organization of a eukaryotic cell, but the functional role of MSCs and their role in complex diseases, such as cancer, are only gradually emerging. In particular, the endoplasmic reticulum (ER)-mitochondria contacts (EMCS) are key effectors of non-vesicular lipid trafficking, thereby regulating the lipid composition of cellular membranes and organelles, their physiological functions and lipid-mediated signaling pathways both in physiological and diseased conditions. In this short review, we discuss key aspects of the functional complexity of EMCS in mammalian cells, with particular emphasis on their role as central hubs for lipid transport between these organelles and how perturbations of these pathways may favor key traits of cancer cells.

## Introduction

Membrane contact sites (MCS) operate as molecular bridges empowering the flow of communication within membrane-bound organelles with specialized cellular functions. Perturbations of MCS are emerging traits of a broad spectrum of diseases, including lysosomal storage diseases, neurodegeneration and cancer (Gómez-Suaga et al., 2018; Peretti et al., 2019; Ballabio and Bonifacino, 2020; Gil-Hernández et al., 2020; Vrijsen et al., 2022). Despite this recognition, the full biological relevance of MCS is only beginning to emerge in its complexity. Possibly all organelles can form heterotypic membrane contact sites in eukaryotic cells. Since the ER is the largest membrane-bound organelle of the eukaryotic cells, it operates as key communication center by making contact with all vital organelles, such as mitochondria, Golgi, endosomes, lysosomes, peroxisomes and the plasma membrane (Wu et al., 2018; Vance, 2020). However, MCS that do not involve the ER, such as lipid droplets (LDs)-peroxisomes, mitochondria-peroxisomes and mitochondria-LDs contacts have also been described recently (Scorrano et al., 2019). Because of their essential role in many aspects of cellular homeostasis, including, but not limited to-energy production, proteostasis and Ca^2+^ signaling**-** ER-mitochondria contacts (ERMES in yeast or EMCS in mammalian cells) have been characterized to a greater extent compared to other inter-organelle appositions.

EMCS are highly dynamic contact regions (usually in the range of 10–80 nm) between the smooth ER and the mitochondrial outer membrane, which are tethered by proteins, without complete fusion of the membranes of both organelles (Scorrano et al., 2019). EMCS regulate numerous biological functions including lipid transfer, Ca^2+^ homeostasis, ROS signaling, autophagy, and mitochondrial dynamics (for a comprehensive discussion about EMCS, we refer to these recent reviews; (Giamogante et al., 2020; Prinz et al., 2020; Wilson and Metzakopian, 2021). The recognition that critical functions of EMCS are dysregulated in pathological conditions such as metabolic diseases, neurodegeneration and cancer, has sparked an increasing interest in these subdomains, which have become a hot topic in biomedical research (Sassano et al., 2017; Rieusset, 2018; Xu et al., 2020; Wilson and Metzakopian, 2021). In cancer cells, perturbations of the signaling functions (i.e., dysfunction) of EMCS by oncogenes reprogram both cancer cell-autonomous (e.g., glucose metabolism, Ca^2+^ fluxes, mitochondria dynamics, redox signaling, cell death) and non-autonomous (inflammation, innate immunity) processes favoring tumor progression (Sassano et al., 2017; Morciano et al., 2018). However, how cancer cell-associated changes of EMCS affect lipid trafficking and reprogramming of lipid metabolism, which is an emerging trait of aggressive cancer, remains poorly understood.

Here we briefly discuss the role of EMCS in Ca^2+^ signaling and lipid transport and highlight how key signaling pathways regulating EMCS functions are harnessed by cancer cells to support the plasticity of their metabolic traits.

### EMCS: The specialized warehouse for Ca^2+^ signaling

**FIGURE 1:**
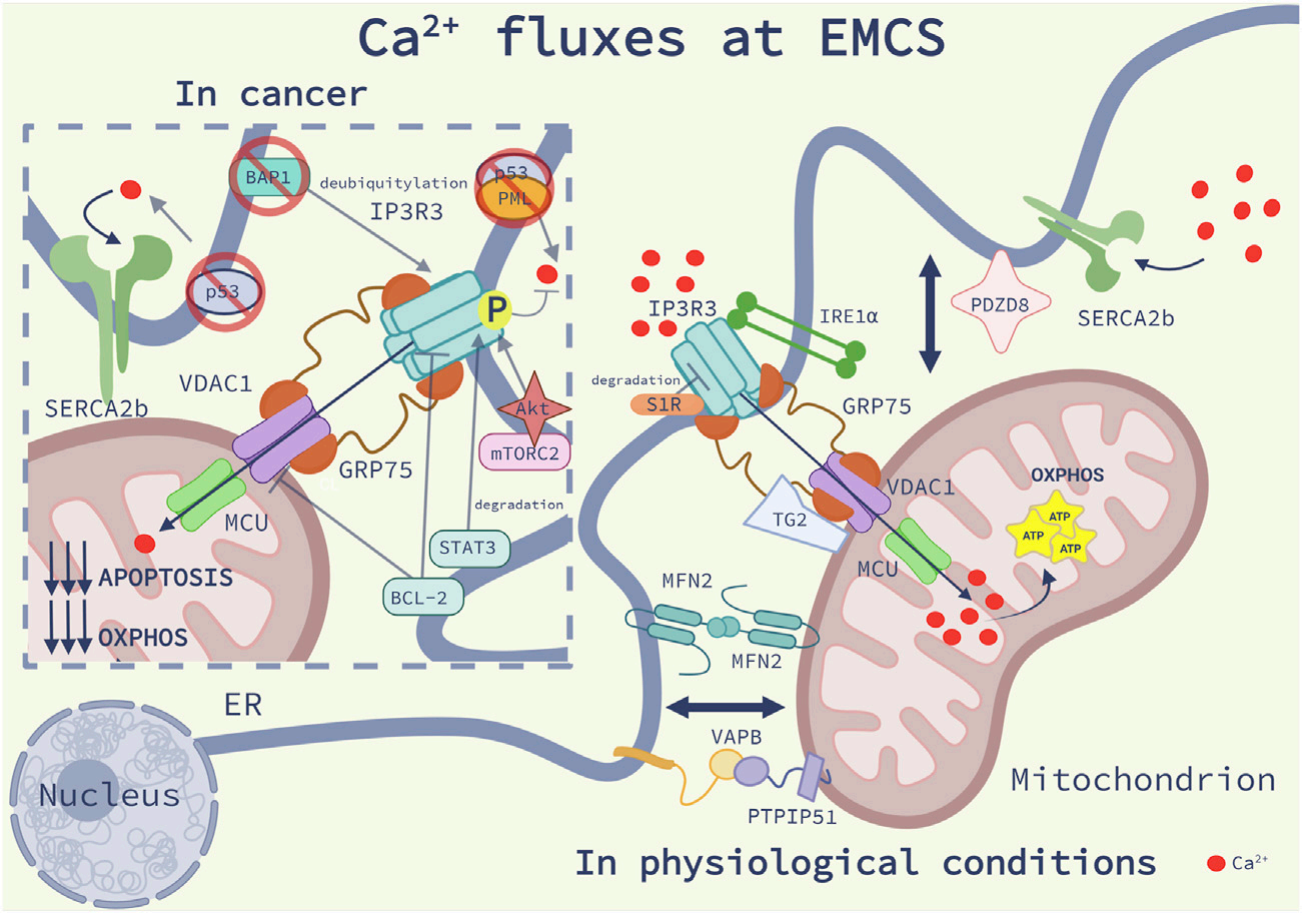
Ca2+ fluxes at EMCS: Schematic representation of the main modulators of Ca^2+^ homeostasis at ER-mitochondria contact sites (EMCS) in physiological conditions (on the right) and in cancer (on the left), as discussed in the main text. On the right side of the image, are represented the main Ca^2+^ regulatory systems at EMCS: the inositol 1,4,5-trisphosphate receptors 3 (IP3R3)- glucose-related regulated protein 75 (Grp75)-voltage-dependent anion channel 1 (VDAC1) signaling complex, delivering Ca2^+^ to the mitochondria, the Ca^2+^ uniporter (MCU), allowing Ca^2+^entry into the mitochondrial matrix, and the sarco/endoplasmic reticulum Ca^2+^ ATPase 2b (SERCA2b), which pumps Ca^2+^ from the cytosol to the ER. Maintenance of mitochondrial Ca^2+^ homeostasis through the integrity of the EMCS, promotes mitochondrial bioenergetics. Sigma-1Receptor (S1R) binds to IP3R3 and decreases its degradation; the ER stress sensor Inositol-requiring enzyme 1 (IRE1α) interacts and brings IP3R3 at EMCS. Transglutaminase type 2 (TG2) binds to Grp75 and promotes mitochondrial Ca^2+^ uptake. The complex formed by the protein tyrosine phosphatase interacting protein 51 (PTPIP51) and the vesicle-associated membrane protein-associated protein B (VAPB), PDZ domain-containing protein 8 (PDZD8) and Mitofusin 2 (MFN2) homodimers favor Ca^2+^ fluxes by regulating the EMCS proximity. On the left side of the image we describe Ca^2+^ dysregulation in cancer. The tumor suppressor p53 at EMCS binds to SERCA pump preventing its ROS-mediated inactivation. P53 interacts with promyelocytic leukemia (PML) protein, promoting the dephosphorylation and inactivation of the proto-oncogene serine/threonine kinase Akt, which together with the Rapamycin complex 2 (mTORC2) phosphorylate and inhibit IP3R3. The proto-oncogene B-cell lymphoma 2 (Bcl-2) interacts and inhibits VDAC1 and IP3R3 suppressing Ca^2+^ fluxes. IP3R3 stability is regulated by the oncogenic signal transducer and activator of transcription 3 (STAT3) which promotes its degradation and the tumor suppressor deubiquitylase BRCA1-associated protein 1 (BAP1), which prevents its ubiquitylation. The mechanisms described here contribute to a reduction of mitochondrial Ca^2+^ import, and consequently to a lower oxidative phosphorylation (OXPHOS) and reduced apoptosis sensitivity.

EMCS play a crucial role in shaping cellular Ca^2+^ fluxes by establishing an intimate interaction between the ER and the mitochondria (Figure 1). The ER is the main Ca^2+^ storage of the cell from which mitochondria take up Ca^2+^, reaching concentration values above those of the bulk cytosol, which in resting conditions are in the range of 100 nM (Rizzuto et al., 1993). The fact that Ca^2+^ enters the mitochondrial matrix via the Ca^2+^ low-affinity mitochondrial Ca^2+^ uniporter (MCU), raised the question of how mitochondria could display such high Ca^2+^ concentration ([Ca^2+^]_mit_) after ER Ca^2+^ release (Rizzuto et al., 1992; Hajnóczky et al., 1995). The discrepancy was solved once in 1998 EMCS were discovered as microdomains of high [Ca^2+^], which is 10-fold higher than the cytosolic ranges (Rizzuto et al., 1998; Csordás et al., 2010). Two main ER-resident proteins are responsible for regulating ER Ca^2+^ homeostasis. The sarco/endoplasmic reticulum Ca^2+^ ATPase (SERCA) pump, which actively pumps Ca^2+^ from the cytosol into the ER lumen to maintain steady-state [Ca^2+^]_ER_ within the 400–1,000 μM range, and the inositol 1,4,5-trisphosphate receptors (IP3R) (Camello et al., 2002). Both transmembrane proteins have been found enriched at the EMCS. In particular, the SERCA2b and the IP3R3 isoforms are preferentially involved in the regulation of Ca^2+^ fluxes at EMCS (Mendes et al., 2005; Lynes et al., 2012). The IP3R channels are responsible for transferring Ca^2+^ from the ER lumen into the mitochondria, by interacting with the mitochondrial voltage-dependent anion channel 1 (VDAC1), located in the mitochondrial outer membrane (OMM), and the ER chaperone glucose-related regulated protein 75 (Grp75) bridging their interaction (Szabadkai et al., 2006).

This molecular machinery shapes Ca^2+^ fluxes between the ER and mitochondria thereby sustaining cellular bioenergetics and mitochondria metabolism (reviewed in (Silva-Pavez et al., 2021), in physiological conditions. Indeed, the transfer of Ca^2+^ from the ER to the mitochondria sustains the activity of Ca^2+^-sensitive mitochondrial dehydrogenases, including pyruvate dehydrogenase (PDH), isocitrate dehydrogenase (IDH) and α-ketoglutarate dehydrogenase (αKGDH) (Hajnóczky et al., 1995). Additionally ER-mitochondria Ca^2+^ transfer regulates vital cellular processes such as autophagy, ER stress and apoptosis (Rowland and Voeltz, 2012; Fan and Simmen, 2019). Given the importance of this Ca^2+^ transferring axis in cellular physiology, EMCS Ca^2+^ dynamics are finely tuned by functional or structural proteins which directly or indirectly modulate Ca^2+^ fluxes (Lee and Min, 2018). Several proteins can affect either the stability, activity or the subcellular localization of the main players of the EMCS Ca^2+^ regulatory system. For example, a notable function of the EMCS-associated ER-chaperone Sigma-1Receptor (S1R), is to attenuate IP3R3 degradation (Hayashi and Su, 2007). More recent studies have underscored that other proteins with enzymatic activities involved in other signaling pathways regulate Ca^2+^ fluxes by localizing at the EMCS. Transglutaminase Type 2 (TG2), by interacting with Grp75, functions as a scaffold for IP3R-Grp75-VDAC1-mediated mitochondrial Ca^2+^transfer (D’Eletto et al., 2018). The ER stress sensor inositol-requiring enzyme 1 (IRE1α), which is one of the main mediators of the unfolded protein response (UPR) (Hetz, 2012) scaffolds the IP3R3 at the EMCS and promotes its ER-mitochondrial Ca^2+^ transfer thereby sustaining mitochondria metabolism under resting conditions (Carreras-Sureda et al., 2019).

As other functions exerted through the EMCS, ER-mitochondria Ca^2+^ homeostasis is also regulated by the distance between these organelles. Disruption of the ER-mitochondria architecture perturbs mitochondrial Ca^2+^ uptake, upon ER Ca^2+^ depletion (Rizzuto et al., 1998; Csordás et al., 2006). In mammalian cells, the ER-mitochondria proximity is modulated by many structural proteins, which guarantee, or disrupt when lost, EMCS integrity and consequently proper Ca^2+^ signaling (we refer to these recent reviews for a broader overview on tethering proteins (Csordás et al., 2018; Lee and Min, 2018; Scorrano et al., 2019; Prinz et al., 2020). The dynamin-like GTPase MFN2, which resides both at the ER and the OMM and is able to form homo-oligomers, is a key regulator of EMCS proximity and Ca^2+^ fluxes between the ER and mitochondria (de Brito and Scorrano, 2008; Filadi et al., 2015; Naon et al., 2016). A well-established tethering complex is formed by the mitochondrial outer membrane protein tyrosine phosphatase interacting protein 51 (PTPIP51) and the integral ER vesicle-associated membrane protein-associated protein B (VAPB) (De Vos et al., 2012). Under conditions causing release of Ca^2+^ from the ER, loss of either VAPB or PTPIP51 is sufficient to blunt the uptake of Ca^2+^ by mitochondria (De Vos et al., 2012). More recently, the PDZ domain-containing protein 8 (PDZD8), a Synaptotagmin-like Mitochondrial lipid-binding Proteins (SMP) domain-containing ER transmembrane protein, was identified as ER-mitochondria tethering protein required to regulate Ca^2+^ dynamics in neurons (Hirabayashi et al., 2017). Interestingly, PDZD8 can also bring the ER and mitochondria in contact with the late endosomes, by interacting with the late-endosomal GTPase Rab7 and the ER transmembrane protein Protrudin (Elbaz-Alon et al., 2020).

While these and other studies (Prinz et al., 2020) support the emerging view that molecular entities of distinct membrane contact sites can be dynamically shared and favor the formation of tripartite organelle contacts, in the following section e focus on discussing how cancer cells reprogram Ca^2+^ signaling at EMCS. The cellular implications of Ca^2+^ in physiological functions have been broadly explained in these studies (Marchi et al., 2017, 2018; Vecellio Reane et al., 2020).

### Dysfunctional Ca^2+^ pathways at EMCS support oncogenesis

Remodeling of the functions, molecular compositions and dynamics of inter-organellar communication, allow cancer cell to rapidly respond to the fluctuating intrinsic metabolic cues and the tumor microenvironmental stress. Over the past years an increasing number of studies indicated that dynamic re-organization of EMCS supports several hallmarks of cancer cells, including, but not limited to, metabolic rewiring, resistance to cell death, migration and invasiveness and responses to inflammatory signals. The full complexity of the role played by MCS in cancer has been discussed in recent reviews (Doghman-Bouguerra and Lalli, 2019; Simoes et al., 2020; Silva-Pavez et al., 2021). Here we briefly discuss some emerging paradigms from the recent literature highlighting the relevance of Ca^2+^ transfer between the ER and mitochondria contacts as critical determinant of cancer cell fate decisions (Figure 1).

Different key tumor suppressors such as p53, the phosphatase tensin homolog (PTEN), promyelocytic leukemia (PML) protein, proto-oncogenes such as the serine/threonine kinase Akt kinase, breast/ovarian cancer susceptibility gene 1 (BRCA1), and various members of the B-cell lymphoma 2 (Bcl-2) family including B-cell lymphoma 2 (Bcl-2), B-cell lymphoma-extra large (Bcl-XL) and BCL-2 ovarian killer (BOK), have been found to localize to ECMS (Thomenius and Distelhorst, 2003; Monaco et al., 2015; Lalier et al., 2021). A major function of their crowded presence at the ER-mitochondria interface is the spatiotemporal modulation of EMCS-resident molecular and signaling complexes, most importantly those formed by the Ca^2+^ releasing IP_3_R channels and the SERCA pump.

Congruently, several studies have revealed that cancer cells are highly dependent on ER-mitochondrial Ca^2+^ transfer for the maintenance of energy balance, the ability to switch between oxidative phosphorylation (OXPHOS), aerobic glycolysis or other metabolic pathways, and for the supply of metabolic intermediates for the biosynthesis of lipid, proteins and nucleic acid required to proliferate and adapt to the metabolically stressed environment (reviewed in (Dejos et al., 2020; Silva-Pavez et al., 2021).

However, the interplay between mitochondrial Ca^2+^ and cellular metabolic pathways is complex and likely shaped by the cancer cell’s specific metabolic requirements and adaptations to nutrients availability. While several cancer subtypes rely primarily on aerobic glycolysis (e.g. Warburg effect), they do not completely shut down mitochondria oxidative phosphorylation. Interestingly, in cells with impaired OXPHOS, mitochondria Ca^2+^ flux through EMCS supports reductive carboxylation and cell survival, by sustaining the activity of the Ca^2+^ sensitive enzyme αKGDH and NADH (Cardenas et al., 2020). On the other hand, the overexpression of mitochondrial calcium uptake 1 (MICU1), a key component of the mitochondrial Ca^2+^ transport system and a negative regulator of MCU, in ovarian cancers drives aerobic glycolysis and is associated to poor survival (Chakraborty et al., 2017). Silencing MICU1 increases mitochondrial Ca^2+^transfer, oxygen consumption by the stimulation of pyruvate dehydrogenase (PDH), which by converting pyruvate to acetyl CoA catalyzes the rate-limiting step of the metabolic fate between glycolysis versus OXPHOS, and results in the inhibition of ovarian cancer growth *in vivo* (Chakraborty et al., 2017). These studies exemplify the complex interplay between mitochondrial Ca^2+^ and metabolic rewiring in cancer through the regulation of Ca^2+^ sensitive matrix dehydrogenases and their metabolites.

Recent studies have revealed a cancer cell dependency on IP_3_R-Bcl-2 interaction for their survival. EMCS-localized Bcl-2 suppresses ER-mitochondria Ca^2+^ fluxes and mitochondrial apoptosis, by interacting through different domains with VDAC1 or the IP_3_R_3_ (the main EMCS-enriched IP_3_R isoform) via specific aa within their BH4 domain (Monaco et al., 2012; Lalier et al., 2021; Rosa et al., 2021). Disrupting the interaction of Bcl-2 and IP_3_R_3_ through a BH4-domain targeting peptide, BIRD2, induces Bax/Bak dependent apoptosis in diffuse large B-cell lymphoma (DLBCL) and chronic lymphocytic leukemia (CLL) cells, through mitochondria Ca^2+^overload driving mitochondrial transition pore opening (mPTP) (Kerkhofs et al., 2021).

The mechanistic target of Rapamycin complex 2 (mTORC2)-Akt axis controls the phosphorylation-mediated inhibition of IP_3_R, which by preventing the transfer of Ca^2+^ from the ER to the mitochondria attenuates mitochondrial apoptosis, while it favors cancer cell’s aerobic glycolysis (Warburg effect) by phosphorylating hexokinase 2 (HK2), recently found as an essential component of EMCS in cancer cells (Betz et al., 2013; Ciscato et al., 2020). This oncogenic mechanism is counterbalanced by a transcriptional independent function of p53. A fraction of p53 localizes at the ER-mitochondria contact sites and establishes a functional interaction with PML (Missiroli et al., 2016). Notably, EMCS-localized PML recruits protein phosphatase 2 A (PP2A) to the complex with the IP_3_R_3_ and Akt. PP2A-mediated dephosphorylation of Akt rescues Ca^2+^ flux from ER to mitochondria and Ca^2+^-dependent apoptosis (Giorgi et al., 2010). Interestingly, disruption of the p53-PML interaction at the ER-mitochondria appositions, by hampering the constitutive ER-Ca^2+−^release, compromises mitochondrial respiration and ATP production, resulting in the stimulation of autophagy through the activation of AMPK (Missiroli et al., 2016). Hence the loss of p53 and consequent removal of PML from these ER membrane subdomains provides a mean to promote tumor growth, by increasing resistance to apoptotic stimuli and increasing adaptation to metabolic stress and anticancer therapy-mediated cellular damage, by stimulating autophagy (Missiroli et al., 2016). Furthermore, EMCS-associated p53 binds to and prevents ROS-inactivation of SERCA. This mechanism maintains ER-Ca^2+^ levels and favors pro-apoptotic Ca^2+^ transfer, which primes cancer cells for mitochondrial apoptosis following oxidative stress or chemotherapy (Giorgi et al., 2015). In contrast, the binding of the thioredoxin-related transmembrane protein (TMX1) to the SERCA2b pump (the housekeeping SERCA isoform at EMCS), results in a lower ER-Ca^2+^ load and low-level constitutive IP_3_-mediated Ca^2+^ release, which rewires metabolism toward aerobic glycolysis and favors tumorigenesis (Lynes et al., 2012).

Beyond phosphorylation and redox-dependent mechanisms, the stability of IP_3_R at the EMCS is controlled by its ubiquitylation status. Recently, the tumor suppressor and deubiquitylase BRCA1-associated protein 1 (BAP1), has been shown to have extranuclear functions in the cytoplasm by localizing at the ER. BAP1 operates as an IP_3_R_3_ deubiquitylating enzyme and supports pro-apoptotic Ca^2+^ signaling under conditions of cellular stress, a mechanism that contributes to the powerful ability of BAP1 in thwarting oncogenesis (Bononi et al., 2017). In particular, BAP1 prevents IP_3_R_3_ ubiquitylation and its subsequent proteasomal degradation (Bononi et al., 2017) by the -box protein L2 (FBXL2), a subunit of the SCF (SKP1-cullin-F-box) ubiquitin-protein ligase complex, which outcompetes PTEN for IP_3_R_3_ binding (Kuchay et al., 2017). IP_3_R_3_ stabilization in breast cancer cells is further regulated by an ER-localized constitutively active form of the oncogenic transcription factor signal transducer and activator of transcription 3 (STAT3). EMCS-associated STAT3 interacts with IP_3_R_3_ and decreases Ca^2+^ transfer to mitochondria by inducing its degradation (Avalle et al., 2019), thereby protecting cancer cells from cell death induced by oxidative damaging agents.

Interestingly, a selective peptide capable to delocalize the mitochondrial outer membrane bound glycolytic enzyme HK2 from EMCS, results in IP_3_R_3_-mediated mitochondrial Ca^2+^overload, killing of patient derived chronic lymphocytic leukemia B cells and reduced cancer growth in mice, without affecting healthy tissues (Ciscato et al., 2020). Together these studies support the notion that targeting molecular mechanisms and mediators of the aberrant ER-mitochondria Ca^2+^ transfer in cancer cells, may represent an effective actionable anti-cancer strategy.

EMCS form a molecular platform for the recruitment of the autophagy machinery (Hamasaki et al., 2013), a fundamental pro-survival process that is often heightened in cancer cells for recent reviews see (Li et al., 2020; Miller and Thorburn, 2021). Hence, altering the expression of EMCS-associated tethers, tumor suppressors or oncogenes, may contribute to control the amplitude of autophagy in cancer cells.

For example, PML was shown to repress pro-tumorigenic autophagy as part of its tumor suppressor activities (Missiroli et al., 2016). In line with this, in response to the loss of PML tumor development is supported by cancer cell-intrinsic autophagy, as a mechanism promoting cell survival during stress conditions (Missiroli et al., 2016). Blockade of the constitutive ER-to-mitochondrial Ca^2+^ transfer lowers OXPHOS, ATP production and results in AMPK activation, which induces autophagy in both normal and cancer cells. However, whereas autophagy promotes the survival of untransformed cells, it may be insufficient to maintain cancer cell viability (Cárdenas et al., 2016). Hence, the functional link between autophagy and dynamics of ER-mitochondria contacts/mitochondrial Ca^2+^ transfer remains complex and it is likely subjected to differential regulation by the network of oncogenes and tumor suppressors formed at the EMCS, the type of cancer cells and their dependency on autophagy for survival and growth.

Growing evidence linking ER-mitochondria appositions and cancer are constantly emerging as more EMCS-resident or associated proteins with growth promoting activity are found to be dysregulated in different cancer types. However, not all these studies have provided conclusive experimental evidence that these proteins favor tumor growth by their specific role as tethers at the ER-mitochondria contact sites.

In conclusion, while future investigations are needed to untangle the role of the growing list of EMCS-associated proteins in cancer cells, the available data suggests that targeting the ER-mitochondria interface may provide novel therapeutic strategies to halt tumor growth and improve therapeutic outcomes.

### Lipid import-export through EMCS

The existence of an internal system to shuttle lipids between organelles was at first considered after the observation that the unique organelle lipid composition differed from that of the main site of bulk lipid production, the ER (Balla et al., 2019). Lipid homeostasis was known to be maintained by vesicular trafficking between organelles but the simple fact that mitochondria were not integrated into these classical routes, fostered the studies of an alternative pathway (Holthuis and Menon, 2014). In 1990 EMCS were isolated and first identified as the site of phospholipid synthesis and non-vesicular transfer between the ER and mitochondria (Vance, 1990). The “crude mitochondria contaminated by ER-derived membranes” fraction was found enriched in lipid synthesis enzymes (Vance, 1990), thus supporting the thought that EMCS served to regulate membranes lipid production and composition in response to cellular demands (Petrungaro and Kornmann, 2019).

A notable function of EMCS is to harbor the synthesis of phosphatidylserine (PS), catalyzed by EMCS-associated phosphatidylserine (PS) synthase 1 and 2 (PSS1; PSS2) (Stone and Vance, 2000). PSS1 converts phosphatidylcholine (PC) into PS while PSS2 synthetizes PS from phosphatidylethanolamine (PE). EMCS synthesis of PS from PSS1 and PSS2 is crucial for mitochondrial PE enrichment. Mitochondria do not directly import PE synthetized in the ER (Vance and Tasseva, 2013; 2013b), but they rather rely on the PS transfer occurring at EMCS, which is then rapidly converted in the mitochondria into PE by PS decarboxylase enzyme (PSD) (Petrungaro and Kornmann, 2019). The active role of the ER-mitochondria platform in determining the abundance of PE in the mitochondria was further confirmed by the massive buildup of PE at EMCS upon inhibition of PSD (Ardail et al., 1991).

Controlling the conversion of PS to mitochondrial PE is essential not only to ensure a local gradient that favors PS transport but also to maintain mitochondrial fitness (Horvath and Daum, 2013). Altering mitochondrial PE levels strongly impairs mitochondrial dynamics, morphology and respiration (Verkleij et al., 1984; Steenbergen et al., 2005; Tasseva et al., 2013; Mårtensson et al., 2017; Zhao and Wang, 2020). Furthermore, PE is a key modulator of autophagy and it is conjugated to LC3 (Atg8 in yeast) through a ubiquitin-like system (Ichimura et al., 2000), suggesting that mitochondrial PE formed through the agency of EMCS may play a role in autophagy-mediated membrane dynamics (Thomas et al., 2018). Mitochondria-derived PE can be transferred back to the ER/EMCS membranes where it is converted into PC by PE-N-methyltransferase (Cui et al., 1993). Curiously, although PC is the most abundant mitochondrial PL (around 40–50%), mitochondria are not able to synthesize it, thus depending entirely on PC import from ER membranes, the site of PC bulk production (Horvath and Daum, 2013). Altering the efficiency of PC transport into the mitochondria impairs cristae formation and destabilizes mitochondrial respiration (Horibata and Sugimoto, 2010; Horibata et al., 2017).

EMCS regulates the homeostasis of another crucial mitochondrial lipid, the anionic phospholipid cardiolipin (CL), which in healthy cells is found in the matrix-facing inner leaflet of the inner mitochondria membrane. Although mitochondria own enzymes to synthesize the precursor of CL, phosphatidic acid (PA), most of the PA converted into CL comes from the ER *via* EMCS (Osman et al., 2011; Potting et al., 2013). CL is involved in the regulation of several physiological processes that occur in the mitochondria. In particular, CL is together with PE a master regulator of mitochondrial respiration (Belikova et al., 2006; Raemy and Martinou, 2014; Hsu et al., 2015). PE and CL bind to and modulate the stability and activity of ETC complexes and respiratory supercomplexes (RSCs) (Acín-Pérez et al., 2008; Böttinger et al., 2012; Horvath and Daum, 2013; Acoba et al., 2020). Specifically, PE and CL bind respectively to complexes I-IV and complexes I-V (Lange et al., 2001; Sun et al., 2005; Sharpley et al., 2006; Schwall et al., 2012; Tasseva et al., 2013; Calzada et al., 2019), regulating mitochondrial bioenergetics and membrane potential (Basu Ball et al., 2018). Consistent with these observations, CL or PE deficient mitochondria exhibit reduced energetic coupling and because of their role in reducing the activity of cytochrome c oxidase (complex IV), diminished mitochondrial membrane potential (Jiang et al., 2000; Böttinger et al., 2012; He et al., 2013; Tasseva et al., 2013; Vance, 2020). The individual components of the mitochondrial respiratory chain assemble into RSCs to stabilize the single components of the ETC and minimize the production of reactive oxygen species. Importantly, while CL by providing a flexible rather than rigid interface between subunits is crucial for the assembly of the RSCs (Fyfe et al., 2001), PE destabilizes it, thus playing an opposite role in their functionalities (Böttinger et al., 2012). In contrast to depletion of CL, disturbance of PE level in mitochondria does not destabilize RSCs but rather favors the assembly of “megacomplexes”, larger RSCs. On the other hand, another study reports that PE does not affect the stability of RSCs, suggesting that PE is required for the activities of ETC complexes while CL regulates both the activity and formation of RSCs (Baker et al., 2016). However, disturbances of mitochondrial levels of PE or CL result in aberrant cristae shape and length (Tasseva et al., 2013; Rampelt et al., 2018; Kondadi et al., 2020).

Beyond maintaining mitochondria respiration, CL plays a pivotal role in initiating apoptosis and the clearance of mitochondria through the process of mitophagy. Under conditions of mitochondrial stress, externalization of CL to the outer membrane serves as an “eat me” signal to target unhealthy mitochondria to the autophagosome, through a direct interaction between CL and LEC3 (Atg8) (Chu et al., 2013). Moreover, in response to cell death stimuli, outer membrane bound CL can serve as a platform for caspase 8 recruitment to the mitochondria. This leads to the caspase-8 mediated cleavage of the BH3 interacting domain death agonist (BID) into its truncated pro-death fragment tBID (Gonzalvez et al., 2008). tBID, together with CL-mediated BAX/BAK oligomerization, fosters mitochondrial cristae remodeling, permeabilization of the OMM and cytochrome c (CYTC) release into the cytosol, triggering apoptosis (Raemy and Martinou, 2014). Moreover, upon ROS production, the CL oxygenase activity of CL-bound CYTC (Kagan et al., 2005) drives the peroxidation of the CL polyunsaturated fatty acid (PUFA) chains, which causes the permeabilization of the OMM and the release of pro-death factors from the mitochondria (Kagan et al., 2005). A close link between EMCS integrity, CL oxidation and apoptosis was suggested in previous studies. In a paradigm of ROS-mediated cell death, weakening the ER-mitochondria contacts by the removal of the Ser/Thr kinase RNA-dependent protein kinase (PKR)-like ER kinase (PERK), another key member of UPR with unconventional tethering function at the EMCS (van Vliet et al., 2018), impaired early CL oxidation and pro-apoptotic cytosolic CYTC release (Verfaillie et al., 2012; van Vliet et al., 2018). Hence, EMCS integrity could serve as an early checkpoint in cell death by promoting CL peroxidation.

EMCS also act as a hotspot for other lipid biosynthetic pathways, which are involved in key cellular processes. The enrichment of cholesteryl esters (CE) and triacylglycerols (TG) synthesizing enzymes, such as acyl-CoA cholesterol acyltransferase-1 (ACAT1) and diacylglycerol acyltransferase 2 (DGAT2) respectively, to EMCS suggest their functional implication in LDs biogenesis (Rusiñol et al., 1994; Stone and Vance, 2000; Stone et al., 2009). Lipid droplets, which bud from the ER membranes, serve as storage sites for neutral lipids and as a supply of fatty acids to mitochondria, to maintain fatty acid oxidation and the tricarboxylic acid (TCA) cycle (Renne and Hariri, 2021). Recently, the existence of a close proximity between LDs, ER and mitochondria, led to the suggestion that EMCS-associated proteins may coordinate the formation of LDs (Benador et al., 2018; Thiam and Ikonen, 2021). In support of this hypothesis, the OMM protein Mitoguardin 2 (MIGA2), which tethers the EMCS by forming a complex with VAPA/B, has been shown to promote the synthesis of TG from non-lipid precursors by linking mitochondria to the ER and LDs (Freyre et al., 2019) and facilitating efficient lipid storage in LDs.

Non-vesicular trafficking of PLs at EMCS (Balla et al., 2019) requires lipid transfer proteins (LTPs), which serve as hydrophilic shuttles to transport PLs between membranes through their hydrophobic cavities (Wong et al., 2019; Prinz et al., 2020). A characteristic feature of LTPs recruited at MCS is the FFAT domain, a short motif made of two phenylalanines in an acidic tract that binds the ER-resident VAP proteins (Murphy and Levine, 2016). Although it is important to distinguish proteins that are structurally in charge of EMCS maintenance from proteins that promote PL transfer functions, it is becoming evident that tethers and LTPs are functionally linked. Several LTPs bind to both the interacting membranes, therefore stabilizing as well the contact. On the other hand, tethers that directly anchor two membranes often display a second motif or domain, which binds lipids and/or lipid binding proteins thus directly or indirectly contributing to the lipid dynamics at EMCS (Scorrano et al., 2019; Wong et al., 2019). The VAPB-PTPIP51 complex has been recently shown to regulate PL transfer at EMCS, in particular the transport of PA (Yeo et al., 2021). Other FFAT-motif proteins are the ER-anchored oxysterol-binding protein-related protein (ORP) 5 and 8, which transfer PS at the ER-PM contact sites (Chung et al., 2015). It has been recently found that ORP5/8 requires the interaction of PTPIP51 and their lipid binding domain [SBP-related lipid-binding (ORD)] to localize at EMCS, suggesting a possible role of this complex in PS transfer at EMCS. Notably, the co-expression of PTPIP51 and ORP5 or 8 reinforces the ER-mitochondria association, therefore functioning as a stabilizing tether (Galmes et al., 2016).

MIGA2 harbors a non-conventional FFAT motif which is required for its interaction with VAPA/B and for the transport of lipid at the EMCS (Freyre et al., 2019). Mitofusin 2 (MFN2) is a master regulator of the contact site proximity (de Brito and Scorrano, 2008; Filadi et al., 2015). Recent evidence shows that MFN2 directly binds and transfers PS across the ER-mitochondria interface (Hernández-Alvarez et al., 2019). Interestingly, PERK is an interactor partner of MFN2 (Muñoz et al., 2013). While the PERK-MFN2 regulatory role under stress conditions has been described, whether both proteins interact to maintain the homeostatic functions of the EMCS remains unclear. Interestingly, PERK is endowed with a lipid kinase activity responsible for diacylglycerol (DAG) phosphorylation and PA production (Bobrovnikova-Marjon et al., 2012). Moreover, PERK interacts with VAPB, although the biological function of their interaction at the EMCS has not been explored yet (Sassano et al., 2021). Together these findings raise the intriguing possibility that PERK might contribute to the regulation of lipid transport at EMCS, a hypothesis that needs experimental confirmation.

**FIGURE 2:**
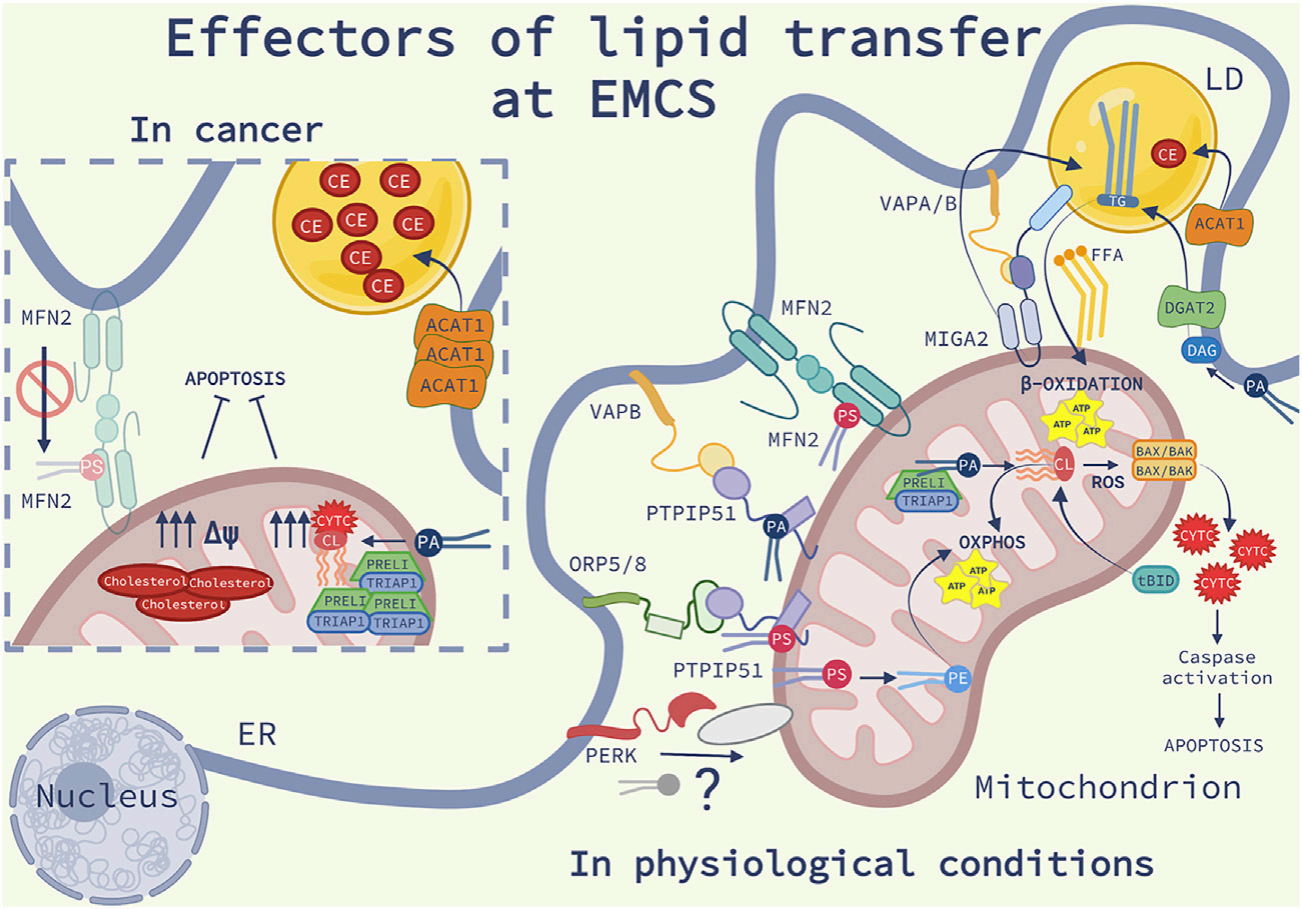
Effectors of lipid transfer at EMCS: Schematic representation of the lipid homeostasis at ER-mitochondria contact sites (EMCS) and lipid droplets (LD), in physiological conditions (on the right) and in cancer (on the left). On the right side of the image, are represented the main lipid transfer proteins (LTP) discussed in the review (see the main text for further explanations); Mitofusin 2 (MFN2) homodimers, bind and transfer phosphatidylserine (PS), the oxysterol-binding protein-related protein (ORP) 5 and 8-protein- tyrosine phosphatase interacting protein 51 (PTPIP51) complex, the vesicle-associated membrane protein-associated protein B (VAPB)- PTPIP51 complex, responsible of the transfer of phosphatidic acid (PA). The Ser/Thr kinase RNA-dependent protein kinase (PKR)-like ER kinase (PERK) is shown to interact with an unknown mitochondrial protein, possibly facilitating phospholipid (PL) transport. In the inner mitochondrial membrane (IMM) PS is converted into PE and PA and transferred into the mitochondrial intermembrane space (IMS) by TP53-regulated inhibitor of apoptosis gene 1 TRIAP1-PRELI complex. PA is converted into cardiolipin (CL), an anionic PL which promotes mitochondrial oxidative phosphorylation (OXPHOS). In response to cell death stimuli, CL interacts with truncated BH3 interacting-domain death agonist (tBID) promoting BAX/BAK oligomerization, release of cytochrome c (CYTC), triggering caspase activation and apoptosis. Mitoguardin 2 (MIGA2)-VAPA/B complex tethers the EMCS to LD and facilitates the production of triglycerides (TG). TG is synthesized into the ER from PA via the diacylglycerol acyltransferase 2 (DGAT2) enriched at EMCS. The enzyme acyl-CoA cholesterol acyltransferase-1 (ACAT1) localized at EMCS synthetizes cholesteryl esters (CE). TG and CE are stored in LD. TG hydrolysis provides free fatty acids (FFA) as fuel for β-oxidation occurring in the mitochondria. On the left panel, some molecular mechanisms involving lipid signaling at EMCS, which are altered in cancer cells, are illustrated: ACAT1 expression is increased, consequently leading to the accumulation of elevated levels of CE into LD; the complex TRIPA1-PRELI is upregulated, PA is converted into CL, impairing the release of CYTC and the sensitivity to apoptosis; cholesterol is highly accumulated into the IMM, affecting the mitochondrial potential (Δψ) and favoring resistance to apoptosis. Deficiency of MFN2 compromises the transferring of PS at EMCS causing ER stress, inflammation, fibrosis and cancer.

Collectively, these observations indicate that a complex regulatory network composed of lipid carrying shuttles, LTPs and multiple tethering complexes, dynamically interact to govern lipid homeostasis, mitochondrial metabolism and cell fate decisions (Figure 2).

### Alterations of the EMCS lipid factory in cancer

Given the crucial role of lipids in mitochondrial metabolism and signaling, it comes as no surprise that cancer cells often present abnormalities in the lipid content by altering their synthesis, storage or transport. In general cancer cells, due to their greater energy demands for survival and growth, increase the *de novo* fatty acid synthesis, necessary for the formation of new structural membranes and as a source of energy (Medes et al., 1953; Simoes et al., 2020).

Since EMCS as discussed above, represent the domains for lipid synthesis and play a pivotal role in cell fate decisions (Balla et al., 2019), they are an ideal platform for cancer lipid remodeling (Figure 2). Cancer cells often present alterations in the levels of inner membrane CL and cholesterol, which may favor resistance to apoptosis or metabolic reprogramming (Kiebish et al., 2008, Kiebish et al., 2009; Monteiro et al., 2013). For example, the tumor suppressor p53 regulates the transport of PA into the mitochondria *via* EMCS (Potting et al., 2013). p53-mediated expression of TP53-regulated inhibitor of apoptosis gene 1 (TRIAP1) and the PRELI complex, was found to promote PA transfer into the mitochondrial intermembrane space (IMS). Disruption of the TRIAP1-PRELI complex reduces levels of PA transfer from the ER into the mitochondrial inner membrane and CL production, which facilitates the release of CYTC and apoptosis (Potting et al., 2013), a mechanism that possibly explains why TRIAP1 is upregulated in multiple myeloma (Park and Nakamura, 2005; Felix et al., 2009).

Additionally, although mitochondria present a low content of cholesterol compared to other organelles, the transfer of cholesterol relies on the ER-mitochondria interconnection to reach the inner mitochondrial membrane (IMM). High cholesterol content affects the IMM permeability in cancer cells, therefore compromising their sensitivity to apoptosis (Sassano et al., 2017; Peretti et al., 2019). In basal state, membrane-bound free cholesterol is converted by the EMCS-localized ACAT1 enzyme into CE and stored in LDs (Puglielli et al., 2001). Breast cancer cells show high levels of ACAT1 and show elevated levels of CE in LDs (Koizume and Miyagi, 2016). Additionally, ACAT1-mediated accumulation of CE in cancer is often associated with proliferation, metastasis and bad prognosis (Koizume and Miyagi, 2016; Li et al., 2016; Stopsack et al., 2017), suggesting that LDs formation is associated with cancer proliferation and progression. Moreover, besides regulating LDs synthesis, in acute myeloid leukemia (AML), cancer cells control autophagy-mediated degradation of LDs through the regulation of the EMCS integrity. In line with this, EMCS have been recently proposed to serve as an autophagic platform supplying free fatty acids (FFAs) from LDs to fuel OXPHOS pathway, necessary for AML cell survival and proliferation (Singh et al., 2009; Bosc et al., 2020).

Cancer cells also alter the expression of various LTPs or EMCS-associated proteins with a role in facilitating lipid transfer between the ER and mitochondria. As mentioned above, PS can be transferred at the EMCS by the ORP5/8 complex. Some studies have correlated the increased expression levels of ORPs to cancer development (Du et al., 2018). In particular, ORP5 expression has been associated with increased cancer cell invasion and metastasis, possibly because of its PS transferring function at EMCS (Koga et al., 2008; Nagano et al., 2015). However, further studies are needed to validate the role of ORP5 in tumor progression. Additionally, other LTPs have been linked to cancer development, such as VAPB and PTPIP51, whose high expression levels are associated with tumor growth in breast cancer (Peretti et al., 2019). Intriguingly, a recent study disclosed MFN2 as a key factor in the development of non-alcoholic fatty liver disease (NAFLD) and liver cancer, due to its role in transferring PS at the EMCS. Hepatic MFN2 deficiency reduces the transfer of PS at EMCS which leads to a decrease in PS synthesis and ER stress, consequently causing inflammation, fibrosis and liver cancer (Hernández-Alvarez et al., 2019).

Together, these studies illustrate the intricate interplay between mitochondrial phospholipid dynamics, relying on the ER-mitochondria communication and mitochondrial homeostasis as a vital node of cancer metabolic adaptation. Nevertheless, further mechanistic analysis is needed to understand how dysfunctional EMCS enable aberrant lipid signaling that supports cancer growth and whether normalizing the EMCS-lipid crosstalk may offer a strategy to halt cancer progression.

## Concluding remarks

Although in the last decade the complex architecture of contact sites has become more and more clear and new family components have been characterized, several outstanding questions still remain to be solved. In general, given the increasing number of newly-identified structural elements part of EMCS, it became important to better understand their mutual regulation and selectivity. Why would organelles display a wide variety of protein complexes regulating the same biological function at EMCS? Are certain protein complexes prioritized and taking over others depending on the cellular stimulus? Do different types of contact sites display different specialized domains? Recent evidence seems to indicate that the majority of proteins targeted to membrane contact sites display a broad range of functions, selectively executed on the bases of their localization, dynamic protein-protein interactions and cellular necessities.

In particular, while studies on Ca^2+^ signaling at EMCS have been quite conclusive and the molecular identities of the main ER-mitochondria Ca^2+^ players have been extensively characterized, molecular components regulating the lipid landscape of the EMCS are largely elusive. How are certain lipids preferentially more prone to vesicular pathways rather than the endomembrane system? Is the assigned route PL-dependent or based on the metabolic state of the cell and cellular stress? Is the close proximity between membranes sufficient for an efficient lipid remodeling at EMCS or are LTPs the “conditio sine qua non”? Answering these biological questions urge a deeper understanding of the lipid dynamics at MCS, requiring complex techniques not fully available nowadays but certainly in rapid progress.

Most importantly, although persuasive evidence indicates that oncogenes or tumor suppressors utilize the EMCS platform to interact with the major protein regulators of Ca^2+^ signaling to promote their survival, the molecular pathways that allow cancer cells to accurately regulate lipid reshuffling at EMCS and their implication in the cancer context are still unclear. Is the remodeling of EMCS proximity also a way for cancer cells to affect lipid cancer metabolism?

Ultimately, a better characterization of Ca^2+^ and lipid signaling and identification of new players at EMCS will facilitate the identification of new targets/signaling pathways hijacked by cancer cells at EMCS, which may help design new therapeutic approaches against cancer.
